# Supplementary figures and images for: Defining the chromatin-associated protein landscapes on Trypanosoma brucei repetitive elements using synthetic TALE proteins
Source: eLife. 2026 Mar 10;14:RP109950. doi: 10.7554/eLife.109950 (PMC12975129; doi:10.7554/eLife.109950)

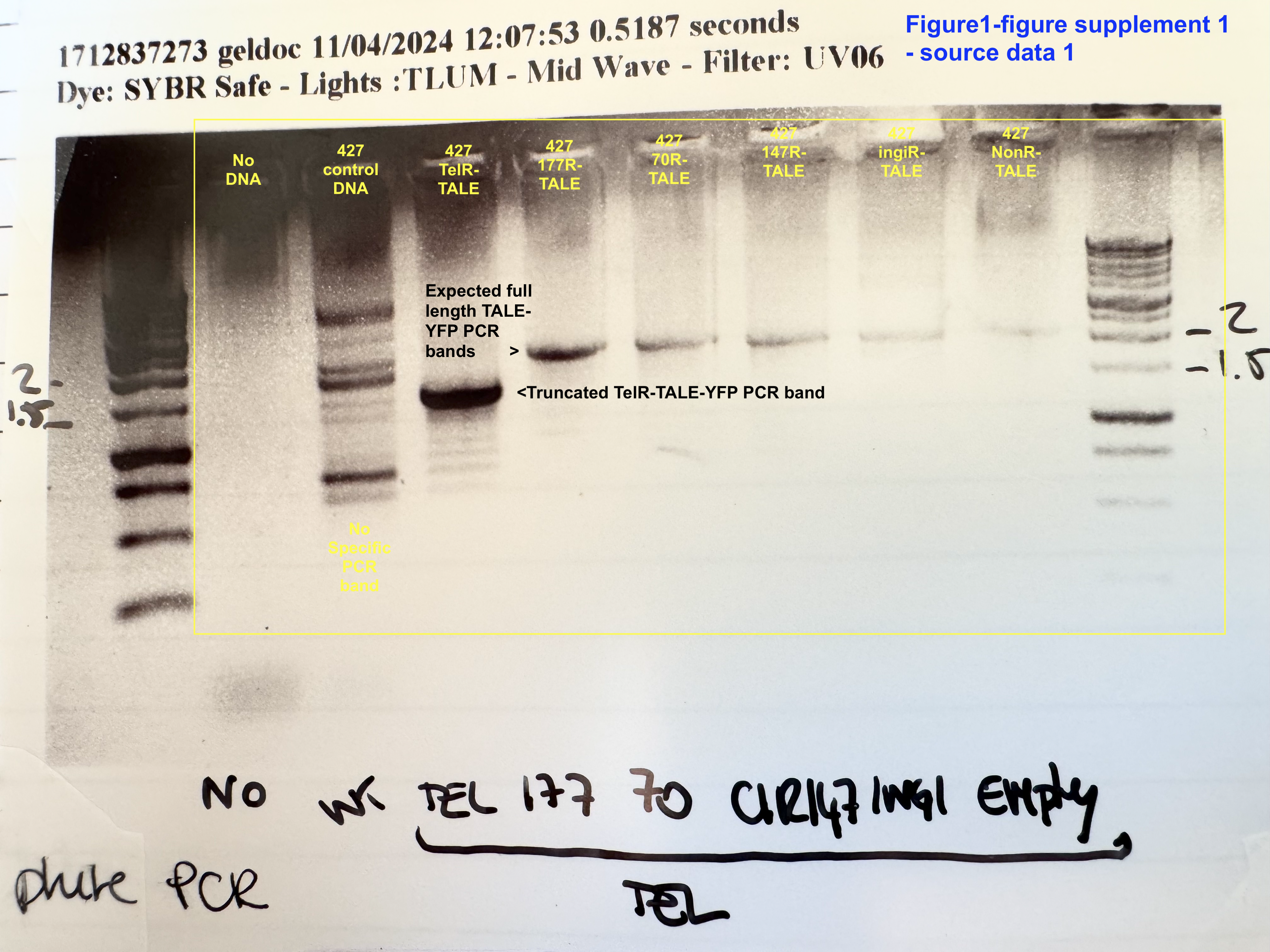

Supplement: Figure 1—figure supplement 1—source data 1. [file elife-109950-fig1-figsupp1-data1.zip › F1_figsup1_sd1_label.tiff]

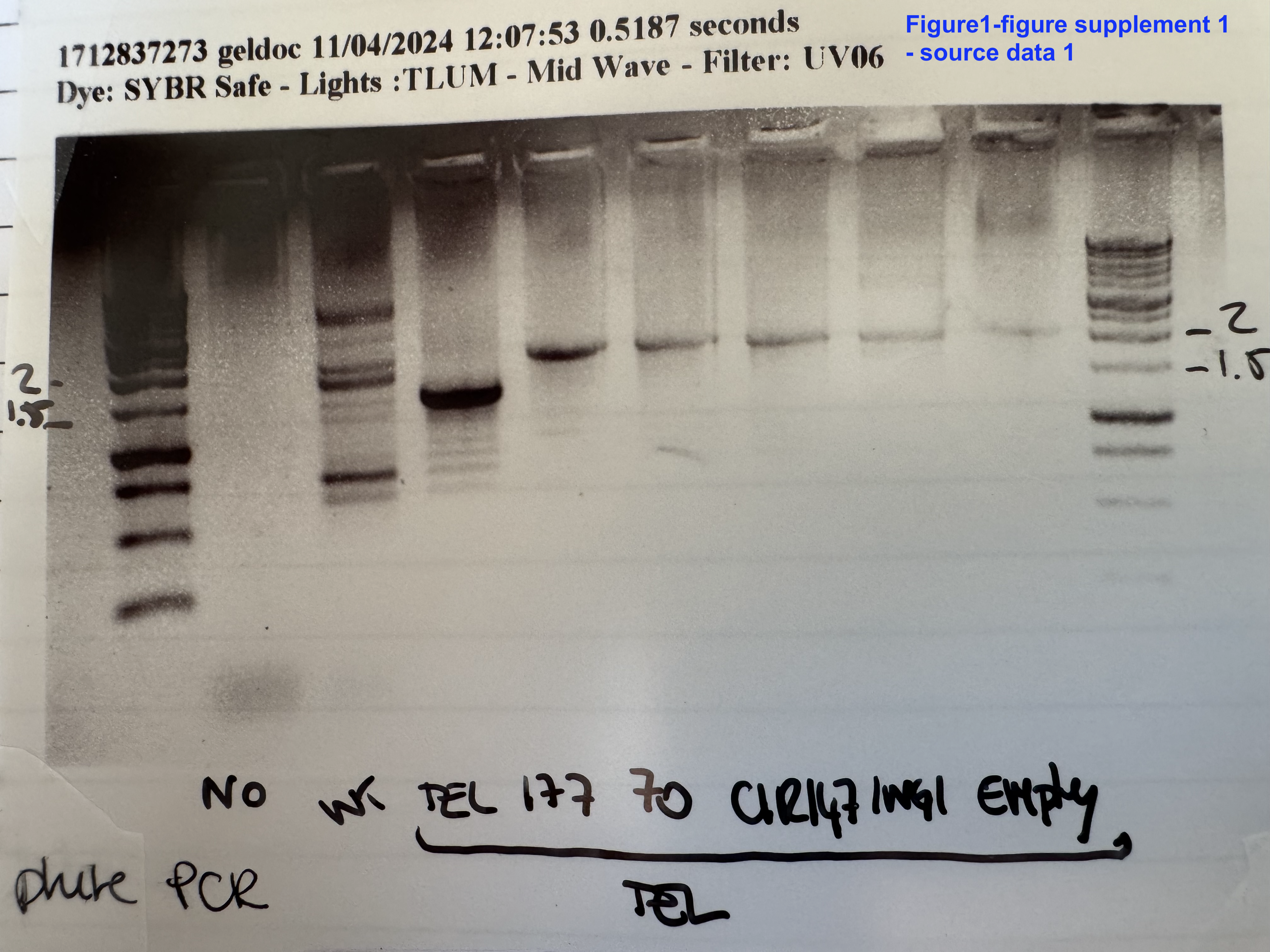

Supplement: Figure 1—figure supplement 1—source data 1. [file elife-109950-fig1-figsupp1-data1.zip › F1_figsup1_sd1_orig.tiff]

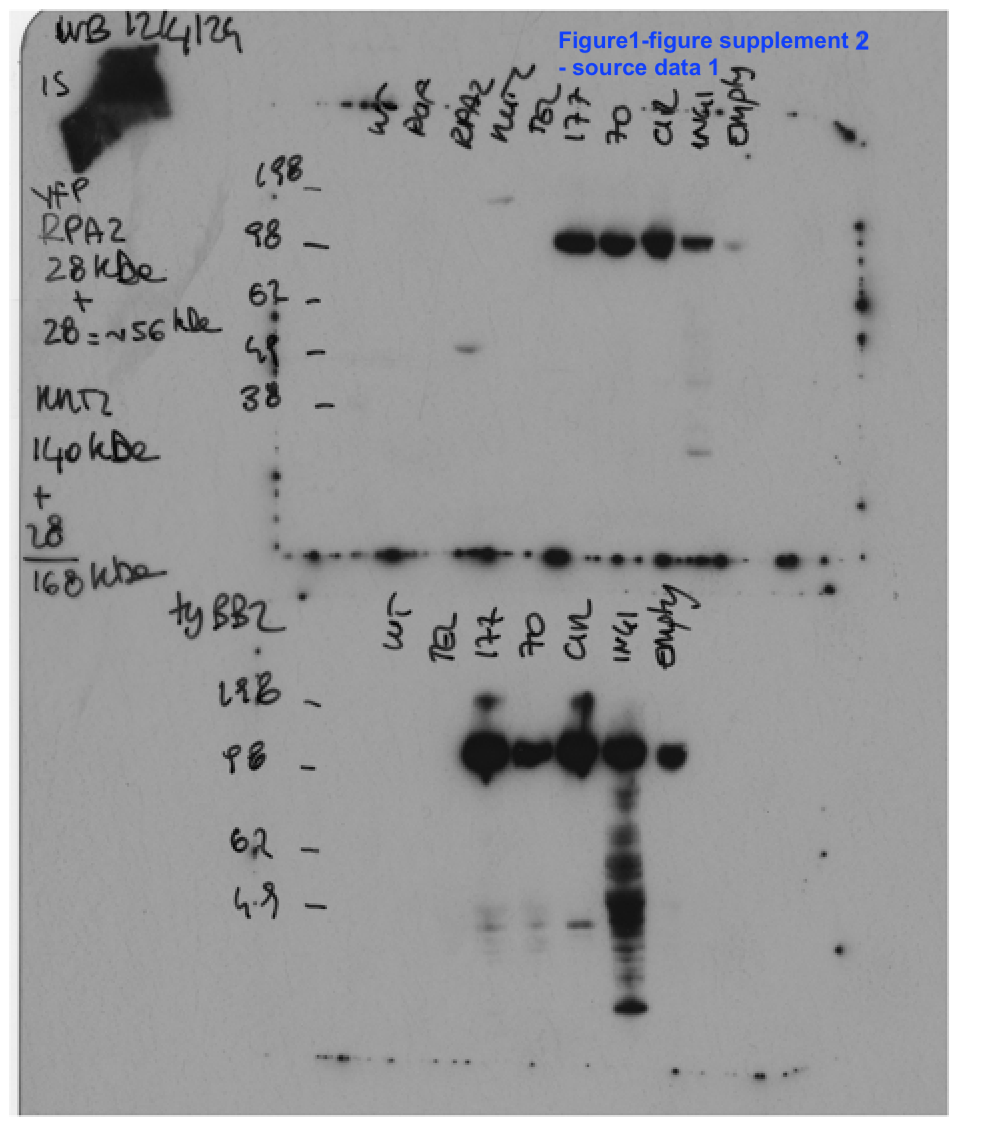

Supplement: Figure 1—figure supplement 2—source data 1. [file elife-109950-fig1-figsupp2-data1.zip › F1_figsup2_sd1_orig.tiff]

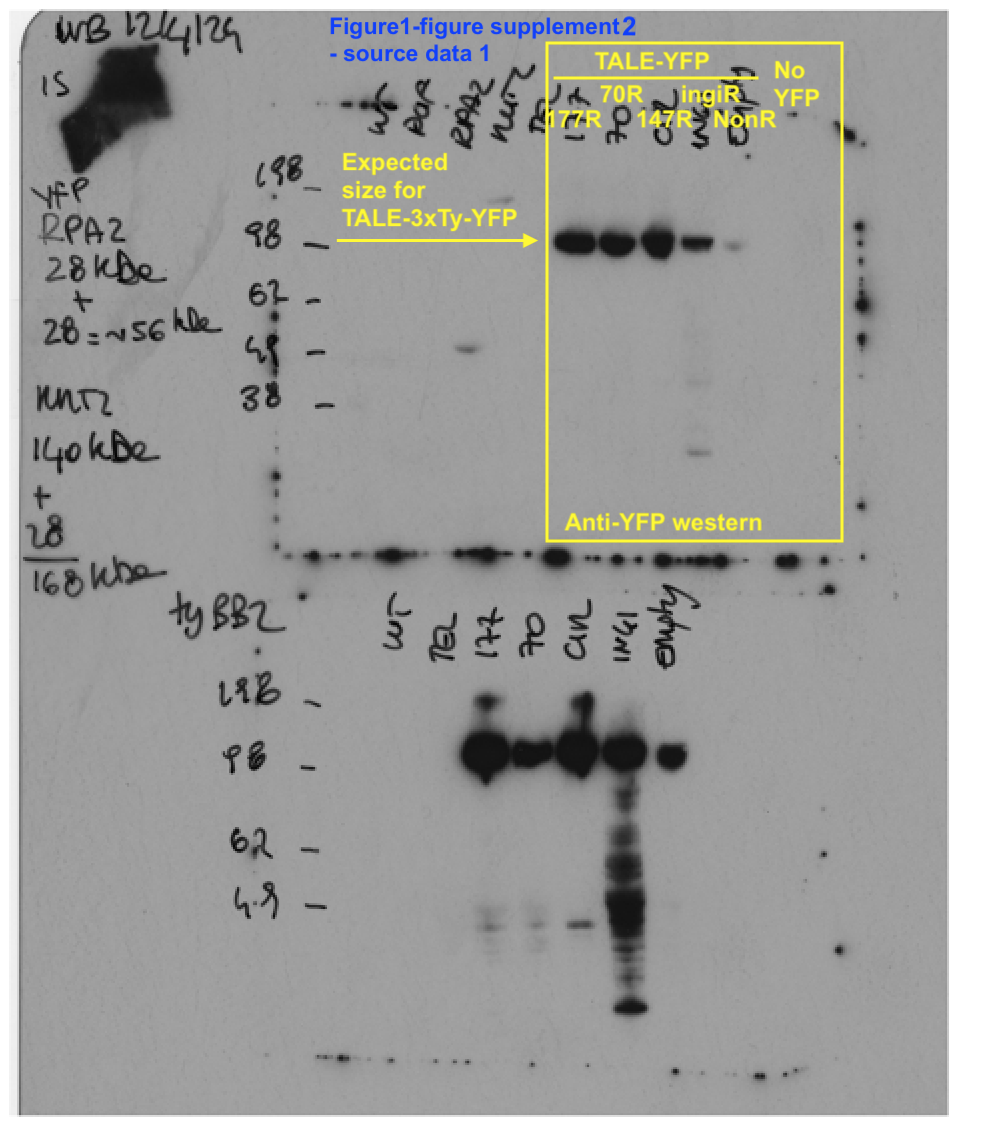

Supplement: Figure 1—figure supplement 2—source data 1. [file elife-109950-fig1-figsupp2-data1.zip › F1_figsup2_sd1_label.tiff]

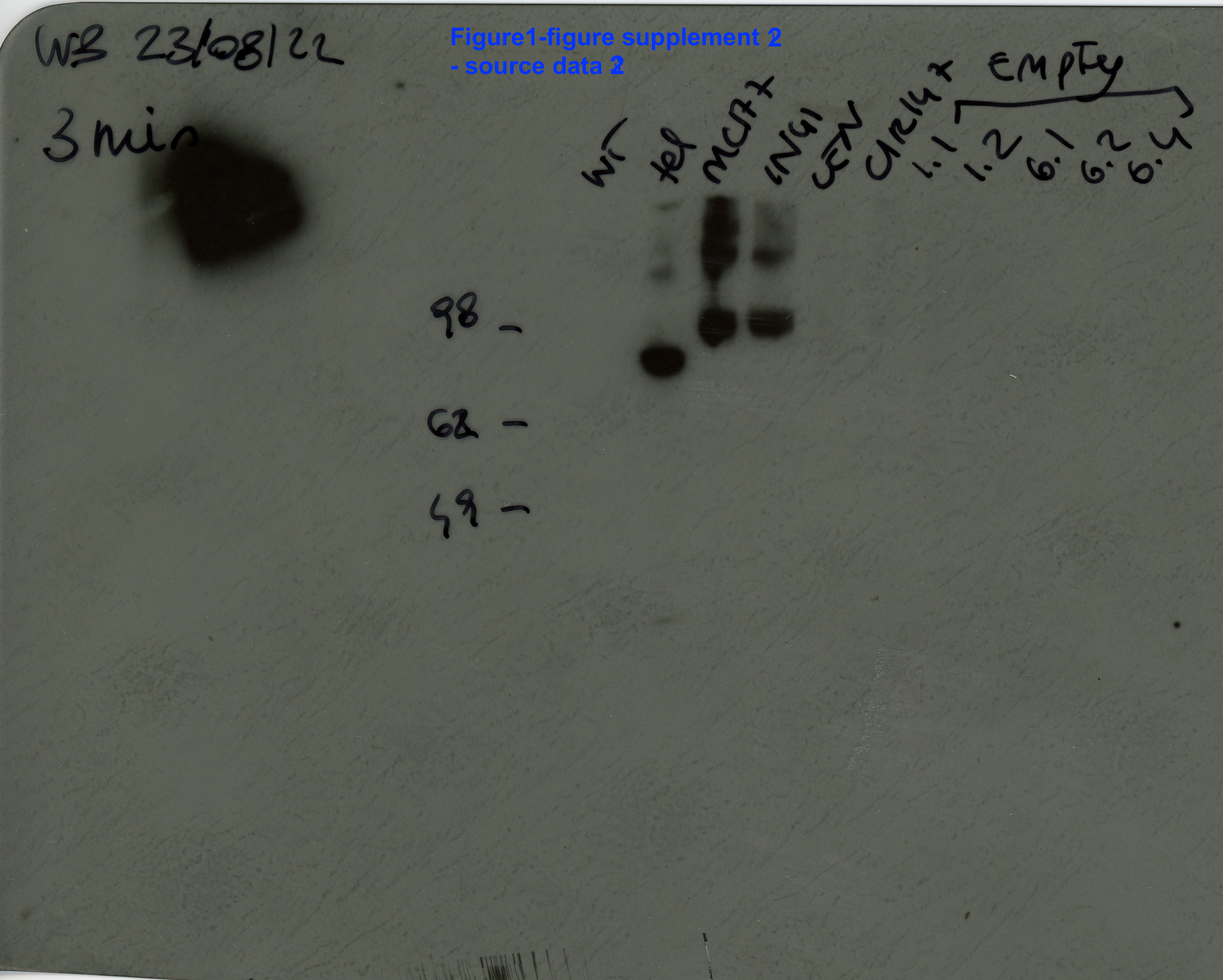

Supplement: Figure 1—figure supplement 2—source data 2. [file elife-109950-fig1-figsupp2-data2.zip › F1_figsup2_sd2_orig.tiff]

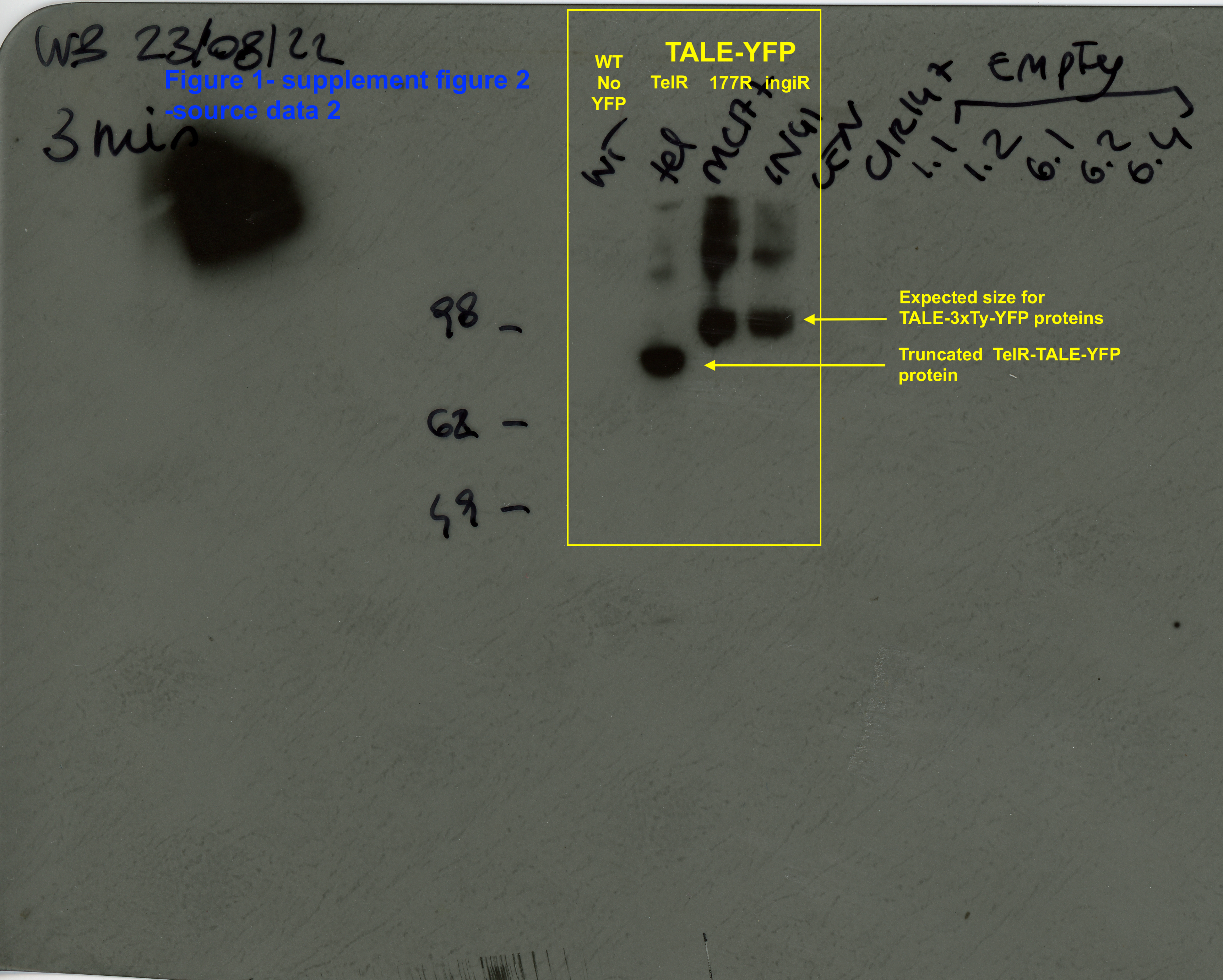

Supplement: Figure 1—figure supplement 2—source data 2. [file elife-109950-fig1-figsupp2-data2.zip › F1_figsup2_sd2.label.tiff]

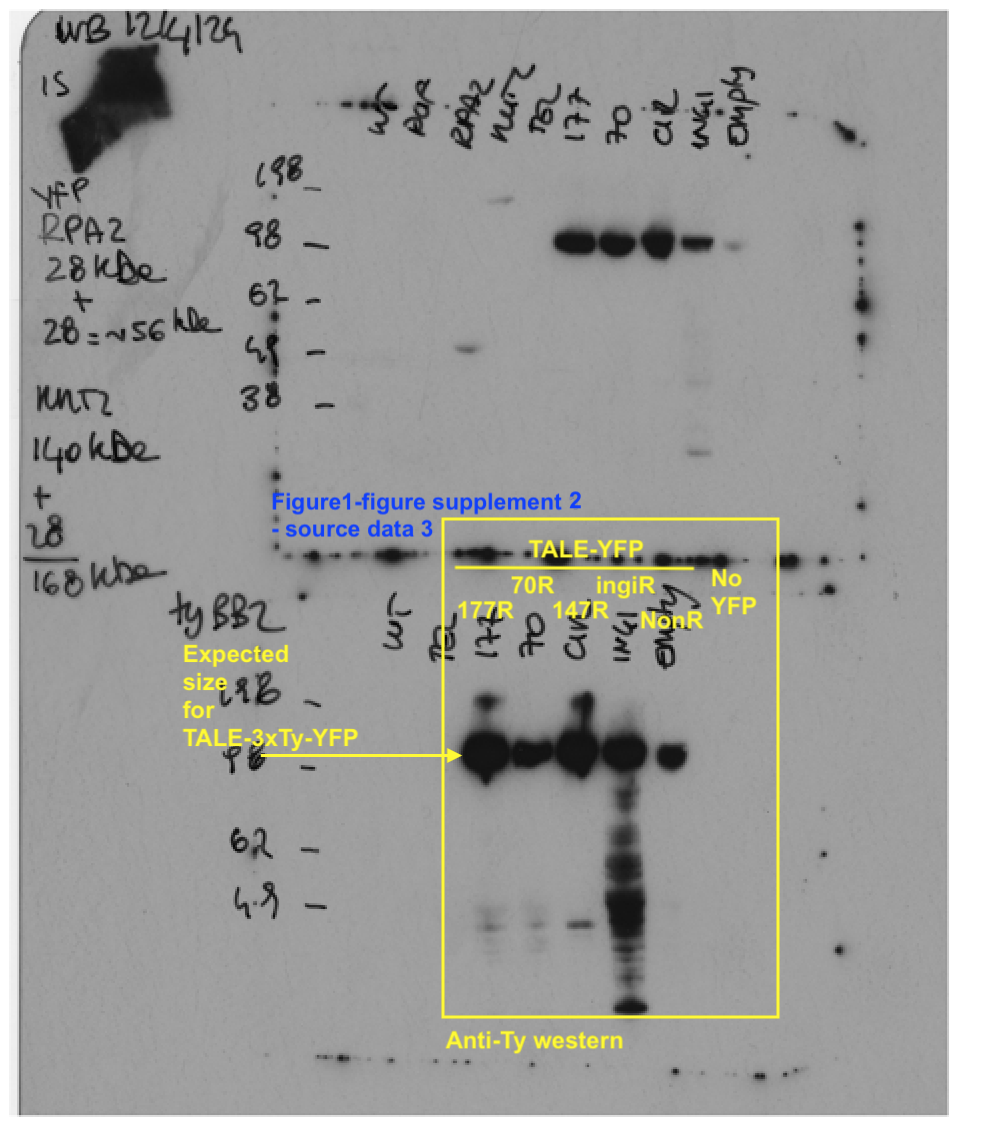

Supplement: Figure 1—figure supplement 2—source data 3. [file elife-109950-fig1-figsupp2-data3.zip › F1_figsup2_sd3_label.tiff]

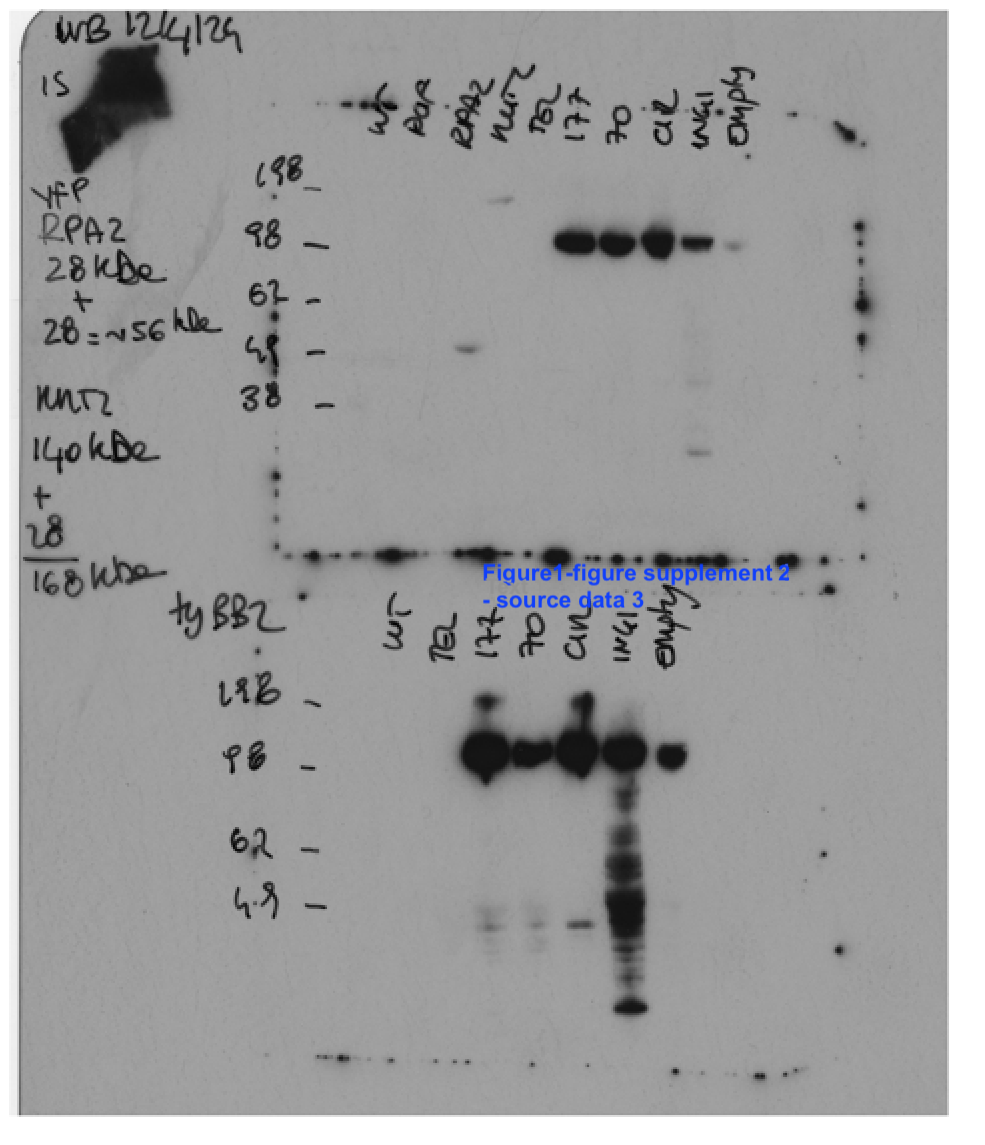

Supplement: Figure 1—figure supplement 2—source data 3. [file elife-109950-fig1-figsupp2-data3.zip › F1_figsup2_sd3_orig.tiff]
